# Maternal Hyperthyroidism in Rats Alters the Composition and Gene Expression of the Matrix Produced In Vitro by Chondrocytes from Offspring with Intrauterine Growth Restriction

**DOI:** 10.3390/metabo12040292

**Published:** 2022-03-26

**Authors:** Fabiana R. Araújo, Bruno M. Bertassoli, Natália M. Ocarino, Amanda M. S. Reis, Juneo F. Silva, Lorena G. R. Ribeiro, Rogéria Serakides

**Affiliations:** 1 Núcleo de Células Tronco e Terapia Celular Animal (NCT-TCA), Escola de Veterinária, Universidade Federal de Minas Gerais, Belo Horizonte 31270-901, Minas Gerais, Brazil; fabianarochaaraujo@gmail.com (F.R.A.); nataliaocarino@gmail.com (N.M.O.); 2 Faculty of Veterinary Medicine, Universidade de Uberaba (UNIUBE), Uberaba 38000-000, Minas Gerais, Brazil; brunobertassoli@gmail.com; 3 Instituto de Ciências Biológicas, Universidade Federal de Minas Gerais, Belo Horizonte 31270-901, Minas Gerais, Brazil; amandamariar@yahoo.com.br; 4 Departamento de Ciências Biológicas, Universidade Estadual de Santa Cruz, Ilheus 45662-900, Bahia, Brazil; juneo.silva@gmail.com; 5 Departamento de Medicina Veterinária, Universidade Federal de Sergipe, Sao Cristovao 49100-000, Sergipe, Brazil; lorena-rocha@academico.ufs.br

**Keywords:** endochondral growth, ossification, pregnancy, thyroid dysfunction, rat

## Abstract

Herein, we aimed to evaluate cultures of femoral chondrocytes from offspring of rats with intrauterine growth restriction (IUGR) induced by maternal hyperthyroidism. Fourteen adult female Wistar rats were divided into two groups, a control group and a group treated with daily L-thyroxine administration using an orogastric tube (50 µg/animal/day) during pregnancy. Three days after birth, the offspring were euthanized for chondrocyte extraction. At 7, 14, and 21 days, viability and alkaline-phosphatase (ALP) activity were assessed using the MTT assay and BCIP/NBT method, respectively, in a 2D culture. Pellets (3D cultures) were stained with periodic acid Schiff (PAS) to assess the morphology and percentage of PAS+ areas. The gene transcripts for *Col2*, *Col10*, *Acan*, *Sox9*, and *Runx2* were evaluated by qRT-PCR. The MTT and ALP-assay results showed no significant differences between the groups. Maternal hyperthyroidism did not alter the chondrocyte morphology, but significantly reduced the percentage of PAS+ areas, decreased the expression of the gene transcripts of *Col2* and *Acan*, and increased *Sox9* expression. Maternal hyperthyroidism in rats alters the composition and gene expression of the matrix produced by chondrocytes from offspring with IUGR. This may be one of the mechanisms through which excess maternal thyroid hormones reduce offspring bone growth.

## 1. Introduction

Thyroid hormones, represented by tri-iodothyronine (T3) and thyroxine (T4), are critical for reproduction, control of energy metabolism, and the development of various organs [1,2]. The fetus is dependent on the placental transfer of maternal thyroid hormones until the 18th to 20th week of gestational age in humans and until the 17th day of gestation in rats [3,4]. Thus, maternal thyroid dysfunction can negatively affect fetal health [1,5].

Thyroid dysfunction can occur at any stage of life, including during pregnancy. Although less prevalent than hypothyroidism, maternal hyperthyroidism has been associated with various adverse effects in both the mother and fetus, such as miscarriage, premature birth, stillbirth, low birth weight, brain developmental anomalies, and other fetal organ abnormalities [2,5].

Our team has studied the changes in the maternal–fetal interface caused by thyroid dysfunctions that result in placental insufficiency and consequently in intrauterine growth restriction (IUGR) [6,7,8,9,10]. IUGR predisposes adults to diseases of the locomotor system, such as osteoarthritis [11]. Therefore, we have also studied the impact of maternal thyroid dysfunction on neonatal organogenesis, particularly on cartilage. Our studies demonstrated that maternal hyperthyroidism reduced bone growth by reducing chondrocyte proliferation and VEGF expression in the cartilaginous epiphysis of neonatal rats [12,13]. This study aims to investigate the synthesis activity of chondrocytes extracted from neonates with IUGR induced by maternal hyperthyroid and cultured in vitro.

Most bones, including long bones, are formed by endochondral ossification, in which a cartilaginous mold develops, which will progressively be replaced by bone in the prenatal life. At birth, endochondral growth continues through the epiphyseal plate and articular cartilage, which are the remnants of the cartilaginous mold. [14,15]. In rats, the primary ossification center is reportedly formed during intrauterine life, while the secondary ossification center is formed during postnatal life; accordingly, in neonates, the entire epiphysis of the long bones is still cartilaginous [16]. Thyroid hormones are essential for skeletal development from fetal life to the onset of puberty. They have key roles in endochondral and intramembranous ossifications, acting directly in skeletal cells but also indirectly, especially via the growth hormone/insulin-like growth factor-1 axis [17,18].

The addition of different concentrations of thyroid hormones to chondrocyte cultures has demonstrated that these hormones inhibit proliferation and induce the hypertrophic differentiation of chondrocytes, with the expression of collagen type X (*Col10*) and metalloproteinase 13 (*Mmp13*), confirming the direct and critical effect of thyroid hormones on the terminal differentiation of chondrocytes and growth-plate morphogenesis [19,20,21,22]. However, to the best of our knowledge, this is the first study to investigate chondrocyte cultures extracted from the femoral epiphysis of rats with IUGR induced by maternal hyperthyroidism. This study aims to evaluate the composition and gene expression of the matrix produced in vitro by chondrocytes extracted from neonatal rats with IUGR induced by maternal hyperthyroid and cultured in vitro. The cell viability of chondrocytes and alkaline-phosphatase activity cultured in a two-dimensional (2D) system were assessed by MTT and 5-bromo-4-chloro-3-indolyl phosphate (BCIP)/nitro blue tetrazolium (NBT) method, respectively. Pellets (3D cultures) were stained with periodic acid Schiff (PAS) to examine the matrix glycosaminoglycans. The gene transcripts of *Sox9*, *Runx2*, *Col2*, *Col10*, and aggrecan were evaluated by real-time quantitative reverse-transcription PCR (qRT-PCR).

## 2. Results

### 2.1. Confirmation of Maternal Hyperthyroidism

**Figure 1:**
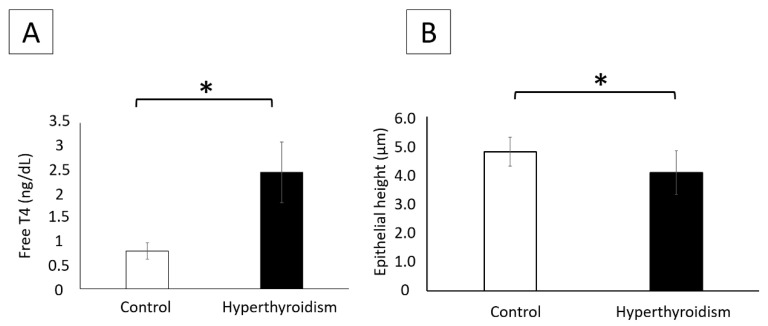
(**A**) Free-T4 plasma concentrations (mean ± standard deviation) of mothers in the control and hyperthyroidism groups. (**B**) Height of the follicular epithelium (μm) of the thyroid (mean ± standard deviation) of control neonates and those exposed to maternal hyperthyroidism * *p* < 0.05.

Hyperthyroidism was confirmed by a significant elevation in plasma concentrations of free T4 when compared with those of the control group (Figure 1A). Unlike the control rats, the T4-treated rats showed clinical signs of agitation and aggressiveness during the handling and passage of the orogastric tube. However, no behavioral test was performed to measure the difference between groups, which is a limitation of this study.

Newborn rats from the control group presented thyroids follicles predominantly lined by cuboidal epithelia and filled with dense colloid, which was sometimes vacuolated. In contrast, the thyroid follicles of newborns exposed to maternal hyperthyroidism were predominantly lined with flattened epithelia, with a significant reduction in the height of the follicular epithelium when compared with that of the control group (Figure 1B).

### 2.2. Confirmation of Reduced Body Weight and Bone Length Caused by Maternal Hyperthyroidism

**Figure 2:**
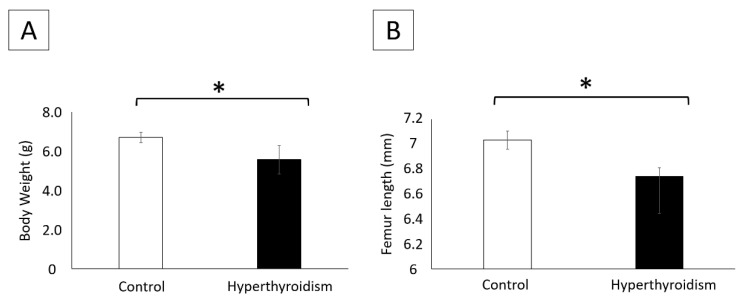
(**A**) Body weight (g) (mean ± standard deviation) of control neonates and those exposed to maternal hyperthyroidism. (**B**) Femur length (mm) (mean ± standard deviation) of control neonates and those exposed to maternal hyperthyroidism * *p* < 0.05.

Neonates from the group of hyperthyroidism-induced mothers showed a significant reduction in body weight and length of the femur when compared with the control (Figure 2A,B). Litter size did not significantly differ between groups (data not shown).

### 2.3. Maternal Hyperthyroidism Does Not Alter Viability and Alkaline-Phosphatase Activity of Offspring Chondrocytes

**Figure 3:**
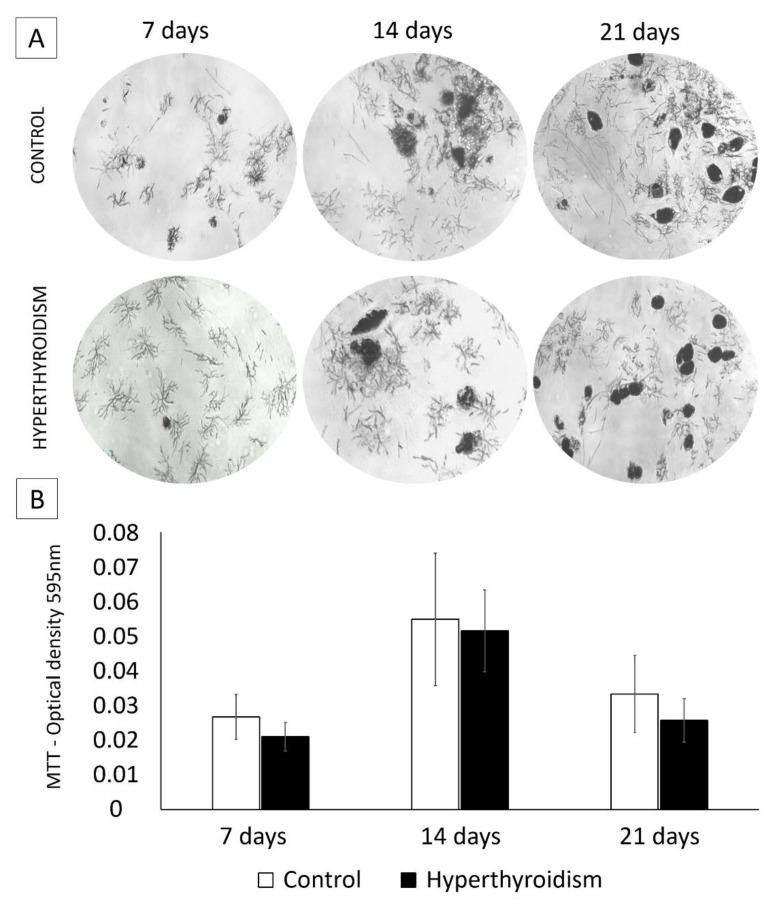
(**A**,**B**) Conversion of MTT to formazan crystals (mean ± standard deviation) in two-dimensional cultures of chondrocytes derived from the femoral epiphysis of control newborn rats and those exposed to maternal hyperthyroidism at 7, 14 and 21 days. Magnification: 25×.

No significant difference was observed between the control group and the group exposed to maternal hyperthyroidism in terms of cell viability at days 7, 14, and 21 (Figure 3).

**Figure 4:**
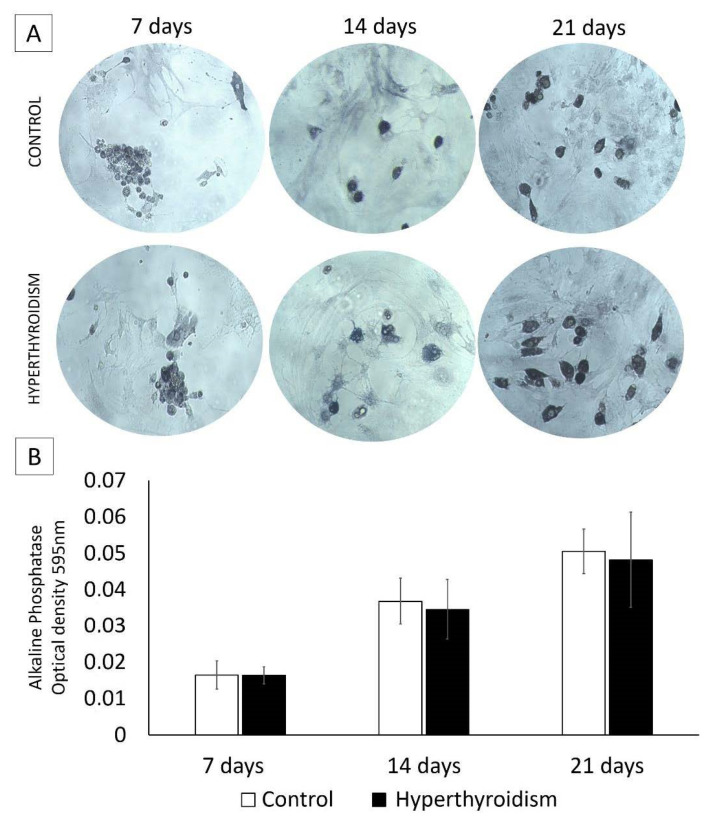
(**A**,**B**) Alkaline-phosphatase activity (mean ± standard deviation) in two-dimensional cultures of chondrocytes extracted from the femoral epiphysis of control newborn rats and those exposed to maternal hyperthyroidism at 7, 14 and 21 days. Magnification: 25×.

No significant difference was observed between the control group and the group exposed to maternal hyperthyroidism in terms of alkaline-phosphatase activity at days 7, 14, and 21 (Figure 4).

### 2.4. Maternal Hyperthyroidism Reduces Glycosaminoglycan Synthesis by Offspring Chondrocytes

**Figure 5:**
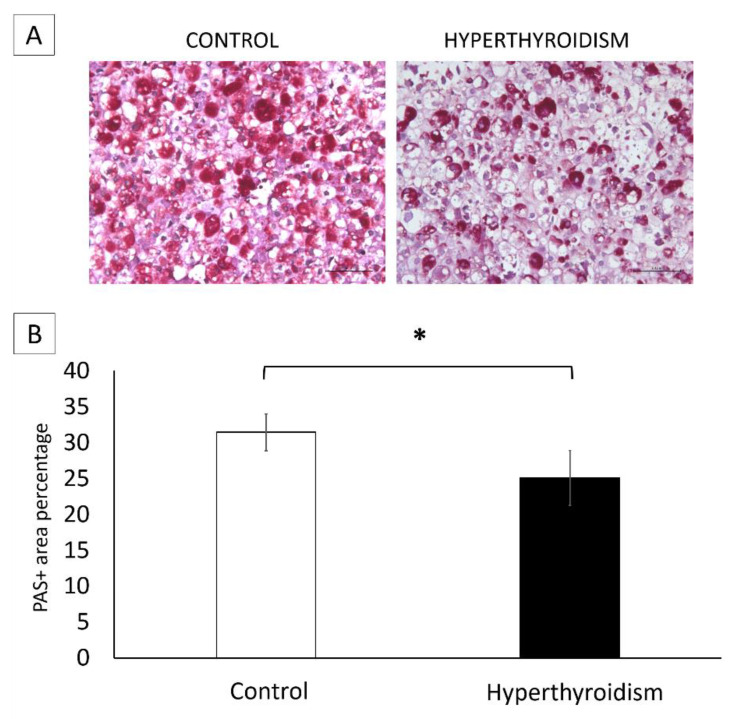
(**A**) Photomicrograph of the 3D culture of chondrocytes derived from the femoral epiphysis of control newborn rats and those exposed to maternal hyperthyroidism, with a reduction in PAS+ areas in the hyperthyroidism group at 21 days. Periodic acid-Schiff staining (PAS). 100×. (**B**) Percentage of PAS+ areas in the 3D culture of chondrocytes derived from the femoral epiphysis of control newborn rats and those exposed to maternal hyperthyroidism at 21 days. * *p* < 0.05.

In both groups, chondrocytes were found to be located in gaps of varying sizes, surrounded by a cartilaginous matrix, with PAS+ and PAS− areas. In addition, the morphological characteristics of chondrocytes in terms of size, cytoplasm, and nucleus were similar between both groups. However, chondrocyte pellets from neonates exposed to maternal hyperthyroidism showed a significant reduction in the percentage of PAS+ areas when compared with the control (Figure 5).

### 2.5. Maternal Hyperthyroidism Reduces the Expression of Col 2 and Acan in Offspring Chondrocytes

**Figure 6:**
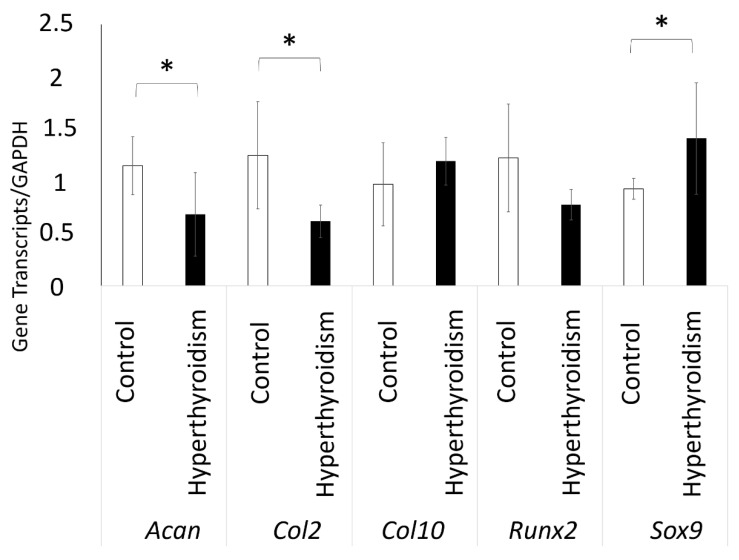
Expression of gene transcripts (mean ± standard deviation) of aggrecan (*Acan*), type II collagen (*Col2*), collagen type X (*Col10*), *Runx2* and *Sox9* by quantitative reverse-transcription PCR (qRT-PCR) in the 3D culture of chondrocytes extracted from the femoral epiphysis of control newborn rats and those with exposure to maternal hyperthyroidism at 21 days. * *p* < 0.05.

At 21 days, the cultures of chondrocytes extracted from newborn rats exposed to maternal hyperthyroidism revealed a significant decrease in the expression of the *Col2* and *Acan* gene transcripts, along with the increased expression of *Sox9*, when compared with the control group. No significant group differences were observed in the expression of *Col10* and *Runx2* (Figure 6).

## 3. Discussion

In the present study, maternal hyperthyroidism was confirmed by a significant increase in the plasma concentrations of free T4 when compared with the control group. Studies that used the same T4 dose during the gestational period were found to satisfactorily induce hyperthyroidism in rats [7,12,13,23]. The body size and blood volume of newborns were extremely small, limiting hormonal performance. Therefore, as previously reported [13,23], the analysis of the newborn thyroid was performed. We observed that the height of the thyroid follicular epithelium was significantly reduced in newborn rats when compared with the control, suggesting a negative feedback effect of excess maternal T4 on the thyroid of the offspring. The reduced height of the thyroid follicular epithelium has been previously reported in neonates exposed to maternal hyperthyroidism [13,23,24]. Under normal conditions, maternal thyroid hormones cross the placenta into the fetal circulation sufficiently to meet fetal requirements. Deiodinases present in the placenta metabolize maternal T4 to T3 for utilization by the fetus, but a significant amount of T4 is also transferred to the fetus [25,26,27]. However, excess maternal T4 can pass to the fetus, reducing serum thyroid-stimulating-hormone (TSH) levels via negative feedback, consequently reducing the height of the thyroid follicular epithelium, as a reduction in TSH has an inhibitory trophic effect on the thyroid [24].

Maternal hyperthyroidism altered the composition and gene expression of the matrix produced in vitro by chondrocytes of the offspring’s femoral epiphysis, presented as a decrease in matrix glycosaminoglycans and evidenced by a decrease in the percentage of PAS+ areas, along with reduced expression levels of *Col2* and *Acan* and elevated *Sox9* expression.

PAS staining allows the assessment of cartilage glycosaminoglycan content in three-dimensional cultures [28,29]. The reduction in the percentage of PAS+ areas in the chondrocyte culture of newborn rats, compared with the control, corresponds with the reduction in *Acan* expression, which is considered the main proteoglycan of the ECM. Thyroid hormones are essential for the synthesis and normal distribution of growth-plate proteoglycans [17,30]. During hypothyroidism, the growth plates of rodents have a higher apparent heparan sulfate content as revealed by Alcian blue critical-electrolyte-concentration histochemistry [31] and the greater immunohistochemical expression of heparan sulfate [32]. In contrast, we have previously reported that excess maternal T4 reduces the intensity of PAS staining in the cartilaginous epiphysis of neonates but does not significantly alter *Acan* expression [23,33]. Therefore, the present study is complementary to the previously published report, quantitatively demonstrating a decrease in the percentage of PAS+ areas and downregulated *Acan* expression in the chondrocyte culture of neonates with maternal hyperthyroidism.

Thyroid hormones stimulate the expression of enzymes that degrade the ECM, such as aggrecanase-2 or *Adamts5* (disintegrin and metalloproteinase with thrombospondin motifs 5) and matrix metalloproteinase13 (*Mmp13*). Thus, the reduction in proteoglycans observed in this study could be attributed to the increased degradation of ECM components [34,35]. Proteoglycans, as well as COL2, are essential for normal cartilage function; therefore, deficiencies in these matrix components reduce chondrogenic differentiation and reduce bone growth [36,37], as observed in the present study.

Several in vitro studies have demonstrated that thyroid hormones, under normal conditions, induce hypertrophic differentiation [19,20,21,22,38]. *RUNX2* and *COL10* expression, as well as alkaline-phosphatase activity, are crucial markers of chondrocyte hypertrophy; therefore, hypertrophic chondrocytes demonstrate enhanced expression of these factors when compared with non-hypertrophic chondrocytes [39]. However, in the present study, neonates with IUGR induced by maternal hyperthyroidism did not show any alterations in the expression of these markers when compared with controls. Ribeiro et al. (2018) [13] have shown that newborn rats exposed to excess maternal T4 present increased thickness of the hypertrophic zone of the growth plate. In the present study, no difference was observed between groups in terms of genotypic characteristics of hypertrophic chondrocytes. Hence, it is possible that maternal hyperthyroidism does not increase the differentiation of hypertrophic chondrocytes but alters the events that cause their death during growth. The increased hypertrophic zone in neonates caused by maternal hyperthyroidism [23] may be attributed to decreased apoptosis of hypertrophic chondrocytes and/or reduced vascular invasion of the hypertrophic zone. In addition, Ribeiro et al. (2018) [13] have demonstrated the reduced expression of VEGF and receptor tyrosine kinase 2 (TIE2), which are angiogenic factors that promote vascular invasion, with a decrease in the hypertrophic-zone thickness, consequently stimulating endochondral bone growth.

In the present study, cultures of chondrocytes derived from the femoral epiphysis of neonates subjected to maternal hyperthyroidism showed increased *Sox9* expression. *SOX9* is a transcription factor that controls different stages of chondrogenesis and regulates several other factors, including the expression of *COL2* and *ACAN* [40]. Accordingly, the expected decrease in the expression of *Acan* and *Col2* would be associated with reduced *Sox9* expression. However, some researchers have previously demonstrated a lack of correlation between the expression of these transcripts in normal and osteoarthritic articular chondrocytes [41,42]. Aigner et al. [42] have evaluated the expression levels of *SOX9* mRNA in normal and osteoarthritic articular cartilage in humans, reporting that expression levels of *COL2* and *SOX9* did not correlate in normal articular cartilage in adults. Furthermore, the authors reported decreased *SOX9* transcription, with a significant increase in *COL2* expression in osteoarthritic chondrocytes. These findings indicate that *SOX9* is not the main transcription factor regulating *COL2* expression. Likewise, there are numerous mechanisms involved in *SOX9* regulation, including post-transcriptional and post-translational modifications and interactions with other gene transcripts, called functional partners [43].

Overexpression of *Sox9* in chondrocytes during embryogenesis can compromise the terminal differentiation of chondrocytes [44,45], with a decrease in *Vegf* expression. VEGF is essential for angiogenesis and, consequently, for endochondral bone growth [46,47,48]. Thus, *Sox9*, by suppressing *Vegf* expression, is an important negative regulator of cartilage vascularization and bone growth from growth plates [46]. Ribeiro et al. (2018) [13] have reported reduced VEGF expression in the cartilaginous epiphysis of newborns exposed to excess maternal T4. Accordingly, we postulated that the increased *Sox9* expression caused by maternal hyperthyroidism could be related to decreased VEGF expression in growth plates [13]. However, the relationship between *Sox9* and *Vegf* in the cartilage growth of animals with IUGR induced by maternal hyperthyroidism needs to be elucidated.

Collectively, our findings revealed that maternal hyperthyroidism alters the composition and gene expression of the matrix produced in vitro by chondrocytes derived from the femoral epiphysis of the offspring with IUGR. In addition, maternal hyperthyroidism decreases the percentage of PAS+ areas, as well as the expression of *Col2* and *Acan* gene transcripts, and increases *Sox9* expression; this might be one mechanism through which excess maternal thyroid hormones reduce the endochondral growth of the offspring with IUGR.

This study did have a few limitations. Unlike the other transcripts studied that remained unchanged or decreased, *Sox9* was the only one that increased in chondrocytes extracted from neonates exposed to maternal hyperthyroidism. This issue needs to be further studied, because initially we expected that there would be a reduction in *Sox9* caused by maternal hyperthyroidism. One of the limitations of this study is that it did not confirm whether the SOX9 protein was also increased. Furthermore, unlike the control rats, T4-treated rats showed clinical signs of agitation and aggressiveness during the handling and passage of the orogastric tube. However, no behavioral test was performed to measure the difference between groups.

## 4. Materials and Methods

All procedures were approved by the Animal Use Ethics Committee (protocol number 216/2019). All animal experiments complied with National Institutes of Health Guide for the Care and Use of Laboratory Animals (NIH Publications No. 8023, revised 1978).

### 4.1. Mating and Thyroxine Administration

Fourteen two-month-old female Wistar rats were housed at a density of four rats per cage on a 12 h light-dark cycle, temperature of 18–22 °C and received food and water ad libitum. The females of all groups were submitted to vaginal cytology and the rats in proestrus were housed in plastic cages with adult male rats for 12 h during the night. The next morning, vaginal smears were performed and gestation was confirmed by the presence of sperm in the vaginal cytology. That day was designated as gestation day 0, and rats were separated, randomly, into individual boxes, comprising the treated (*n* = 7) and control groups (*n* = 7).

Treated rats received daily L-thyroxine (Sigma-Aldrich, St. Louis, MO, USA), administered using an orogastric tube, at a dose of 50 μg/animal/day, diluted in 5 mL of distilled water according to previously established protocols [13,23,49]; this treatment was performed throughout the gestation period and for three days of lactation. Females in the control group received the same volume of distilled water using an orogastric tube.

Three days after birth, neonates from each mother were euthanized. After intraperitoneal anesthesia with ketamine (100 mg/kg) (Sintec, São Paulo, Brasil) and xylazine (10 mg/kg) (Sintec, São Paulo, Brasil), rats were euthanized by cardiac puncture.

### 4.2. Dosage of Maternal Free T4 and Histomorphometric Analysis of the Neonate Thyroids

On the third day of lactation, when chondrocyte extraction from neonates was performed, the mothers’ plasma was collected to measure free T4, which was performed using the chemiluminescence ELISA technique (sensitivity: 0.4 ng/dL) with commercial kits according to the manufacturer’s instructions (IMMULITE, Siemens Medical Solutions Diagnostics, Malvern, PA, USA).

The thyroids from neonates were fixed in 10% formaldehyde for 24 h and later processed and embedded in paraffin. 3 μm-thick sections were stained using the hematoxylin-eosin technique. The height of the epithelium was measured in 20 follicles at four points equidistant from the follicle with the aid of the ImageJ 1.52 k program (National Institute of Health, Bethesda, MD, USA).

### 4.3. Body Weight and Femur Length Measurement

At the time of chondrocyte isolation, seven neonates per group were weighed and the femurs were dissected for length measurement with the aid of a digital pachymeter.

### 4.4. Isolation and Culture of Chondrocytes

After euthanasia, the skin of the pelvic limbs was sterilized. The femurs were dissected from the muscle and connective tissues with sterile instruments, using a laminar flow, washed in sterile phosphate-buffered saline (PBS; 0.15 M), and the cartilage was harvested from the proximal end of the femur. For chondrocyte isolation, the cartilage fragments were washed with PBS (0.15 M) and incubated for 12 h in a solution containing Dulbecco’s Modified Eagle Medium-low glucose (DMEM-Low glucose) (Gibco, Invitrogen, NY, USA), 5% fetal bovine serum (Gibco, Invitrogen, NY, USA), and 4% collagenase type I (Sigma-Aldrich, St. Louis, MO, USA). Next, the chondrocyte suspensions were centrifuged at 1400 rpm for 10 min, washed with 0.15 M PBS, and centrifuged again. Then, cells were resuspended and cultured in T75 bottles with chondrogenic medium (low glucose DMEM plus gentamicin (60 mg/L), penicillin (100 IU/mL), streptomycin (100 mg/mL) and amphotericin B (25 mg/L), 1 % fetal bovine serum, ascorbic acid (50 μg/mL), dexamethasone (10^−7^ M), insulin (6.25 μg/mL), transferrin (6.25 μg/mL), pyruvate (1 mM), and bovine serum albumin (1.25 μg/mL) and maintained in an oven at 37 °C and 5% CO_2_. After 24 h, cultures were washed with PBS solution (0.15 M) to remove non-adherent cells. The medium was replaced twice weekly. In the second passage, the cells were transferred to 24-well plates (2 × 104 cells/well) and were cultured in a 2D system. Additionally, cells were transferred to 15 mL conical plastic tubes (1 × 106 cells/tube) and cultured either in pellets or the 3D system, according to each test. The sampling unit consisted of a pool of cells from four newborns of each mother, totaling seven repetitions per group for each of the assays described below.

### 4.5. Cell Viability Test (Conversion of MTT to Formazan Crystals)

At 7, 14, and 21 days, chondrocyte cultures in a 2D system were washed with PBS solution (0.15 M). Chondrogenic medium (210 µL) was added to 170 µL of MTT (Invitrogen, Carlsbad, CA, USA). The plates were incubated for 2 h (37 °C in 5% CO_2_). A volume of 210 µL of SDS-10% HCl was added, then 100 µL from each well was transferred to a 96-well plate and read on a spectrophotometer at 595 nm wavelength.

### 4.6. Alkaline-Phosphatase Activity by BCIP/NBT Method

At 7, 14, and 21 days, chondrocyte cultures in a 2D system were washed with PBS solution (0.15 M). Each well received the BCIP/NBT solution (1 mL of alkaline-phosphatase buffer, 4.4 mL of nitro-blue tetrazolium chloride (NBT) and 3.3 mL of 5-bromo-4-chloro-3′-indolyl phosphate p-toluidine salt (BCIP) and was incubated for 2 h (37 °C in 5% CO_2_). After, 210 µL of 10% SDS-HCl solution was added to each well and the plates were incubated for 12 h at 37 °C and 5% CO_2_. A total of 100 µL from each well was transferred to 96-well plates and measured using a spectrophotometer at 595 nm wavelength.

### 4.7. Glycosaminoglycan Analysis

At 21 days, chondrocyte pellets were fixed with 4% paraformaldehyde and processed for paraffin inclusion. Serial sections of the pellet were stained with PAS to examine matrix glycosaminoglycans. The percentages of PAS-positive and PAS-negative areas were determined. Five fields from each slide, totaling 25 fields, were photographed at 40× magnification. The images were analyzed by counting points over PAS-positive areas and PAS-negative areas using Image J software (National Institute of Health). The number of points was converted to a percentage.

### 4.8. Evaluation of Gene Transcript Expression

For RT-qPCR, chondrocyte cultures in a 3D system were used at 21 days for the evaluation of the gene expression of aggrecan (*Acan*), *Col2*, *Col10*, *Runx2*, and *Sox9*. RNA extraction was carried out with TRIzol and cDNA synthesis and PCR reactions followed the instructions of the manufacturer of SuperScript III Platinum Two- Step qPCR Kit with SYBR Green (Invitrogen, Carlsbad, CA, USA). Gene expression was calculated through the 2-ΔΔCt method. The sample values were calculated in relation to the GAPDH Ct values. The primers were as follow: forward 5′-CAACTCCCTCAAGATTGTCAGCAA-3′ and reverse 5′-GGCATGGACTGTGGTCATGA-3′ for *Gapdh* (reference sequence: NM_002046); forward 5′-CACACGCTACACACTGGACT-3′ and reverse 5′-TCACACTGGTGGAAGCCATC-3′ for aggrecan (reference sequence: NM 022190.1); foward 5′-GTTCACGTACACTGCCCTGA-3′ and reverse 5′-AAGGCGTGAGGTCTTCTGTG-3′ for *Col2* (reference sequence: NM_012929.1); foward 5′-GAAACAGGTGTCTGACTTAC-3′ and reverse 5′-TACTTCCAGTGGAATAGAAG-3′ for *Col10* (reference sequence: XM_001053056.3); foward 5′-CCCGATCTGAAGAAGGAGAGC-3′ and reverse 5′-GTTCTTCACCGACTTCCTCCG-3′ for *Sox9* (reference sequence: NM_080403.1); foward 5′-GCGTCAACACCATCATTCTG-3′ and reverse 5′-CAGACCAGCAGCACTCCATC-3′ for *Runx2* (reference sequence: NM_004348) [50].

### 4.9. Statistical Analysis

The study followed a randomized design. Data values are presented as the mean ± standard deviation for each variable. The statistical assumptions of normality and homoscedasticity of variances were evaluated by the Shapiro–Wilk and Brown–Forsythe tests, respectively. Birth weight, femur length, and glycosaminoglycan analysis parameters that met these assumptions were subjected to the Student’s *t*-test.

Results of MTT, ALP, and qPCR assays were analyzed with the Mann-Whitney test. All analyses were performed by the GraphPad Prism program (GraphPad Software, Inc., San Diego, CA, USA), considering a significance level of 5% for all variables.

## Data Availability

The authors confirm that the data supporting the findings of this study are available within the article. Further enquiries can be directed to the corresponding author.
